# Cardiovascular risk factor assessment after pre-eclampsia in primary care

**DOI:** 10.1186/1471-2296-10-77

**Published:** 2009-12-08

**Authors:** Marie-Elise Nijdam, Monique R Timmerman, Arie Franx, Hein W Bruinse, Mattijs E Numans, Diederick E Grobbee, Michiel L Bots

**Affiliations:** 1 Julius Center for Health Sciences and Primary Care, University Medical Center Utrecht, Utrecht, the Netherlands; 2 Department of Obstetrics and Gynaecology, St Elisabeth Hospital, Tilburg, the Netherlands; 3 Department of Obstetrics and Gynaecology, University Medical Center Utrecht, the Netherlands

## Abstract

**Background:**

Pre-eclampsia is associated with an increased risk of development of cardiovascular disease later in life. It is not known how general practitioners in the Netherlands care for these women after delivery with respect to cardiovascular risk factor management.

**Methods:**

Review of medical records of 1196 women in four primary health care centres, who were registered from January 2000 until July 2007 with an International Classification of Primary Care (ICPC) code indicating pregnancy. Records were searched for indicators of pre-eclampsia. Of those who experienced pre-eclampsia and of a random sample of 150 women who did not, the following information on cardiovascular risk factor management after pregnancy was extracted from the records: frequency and timing of blood pressure, cholesterol and glucose measurements - and vascular diagnoses. Additionally the sensitivity and specificity of ICPC coding for pre-eclampsia were determined.

**Results:**

35 women experienced pre-eclampsia. Blood pressure was more often checked after pregnancy in these women than in controls (57.1% vs. 12.0%, p < 0.001). In 50% of the cases blood pressure was measured within 3 months after delivery with no further follow-up visit. A check for glucose and cholesterol levels was rare, and equally frequent in PE and control women. 20% of the previously normotensive women in the PE group had hypertension at one or more occasions after three months post partum versus none in the control group. The ICPC coding for pre-eclampsia showed a sensitivity of 51.4% and a specificity of 100.0%.

**Conclusion:**

Despite the evidence of increased risk of future cardiovascular disease in women with a history of pre-eclampsia, follow-up of these women is insufficient and undeveloped in primary care in the Netherlands.

## Background

Women with a history of pre-eclampsia (PE) during pregnancy have an increased risk of chronic hypertension later in life as compared to nulliparous women and women with an uncomplicated pregnancy. In addition, a number of large cohort studies have indicated an increased risk of several other cardiovascular conditions such as overweight and diabetes in later years among women who experienced PE or eclampsia during their pregnancy [1-5]. We recently showed that women with a high blood pressure in pregnancy had a 57% increased risk of developing coronary calcifications later in life [6]. Furthermore, in a recent meta-analysis it was shown that a history of PE increases the risk of future ischemic heart disease, stroke and other vascular diseases [7]. The primary care physician may play an important role in the follow-up of these women after pregnancy, in terms of early identification of high risk subjects and early initiation of treatment of hypertension and other risk factors for CVD. As yet, specific guidelines or protocols for general practitioners (GPs) on cardiovascular risk factor management of women with a pregnancy complicated by PE do not exist. In fact, in the Netherlands routine blood pressure measurements are made, and only when the GP considers a patient at high risk of having elevated blood pressure. Previous pre-eclampsia is generally not considered as identifier of such a high risk group. In addition, it is not known how GPs in the Netherlands care for women who experienced a hypertensive disorder during their pregnancy after the child is born.

We set out to investigate the extent of cardiovascular risk factor management in general practice in women with a history of PE. Additionally we compared the International Classification of Primary Care (ICPC) coding for these disorders in the GPs registries with clinical information from medical records to determine the usefulness of ICPC codes in research.

## Methods

### Study population

The study population comprised all inhabitants of Leidsche Rijn, a geographically well defined new suburb near Utrecht, The Netherlands. Studies with routine healthcare data from this population are carried out in the "Utrecht Health Project" which is a research infrastructure closely linked to primary health care in Leidsche Rijn [8]. The primary health care is delivered by 4 general practices, comprising approximately 18 GPs. The GPs use electronic medical dossiers (EMD) and as part of their usual practice all conditions of the patients are classified according to the International Classification of Primary Care. The GPs and assistants are trained and supported in computerized recording and coding [9]. The study has been approved by the Medical Ethics Committee of the University Medical Centre Utrecht. All participants gave written informed consent. The electronic medical dossiers over the period January 1 2000 - July 12 2007 were used to define our study population.

We identified ICPC codes potentially dealing with pregnancy (additional file 1). The corresponding medical charts were manually reviewed to determine the exact number of pregnancies and to identify potentially wrongly coded records. We confirmed the presence of an ongoing pregnancy when, in addition to the ICPC code, information was available in the records indicating pregnancy (e.g. confirmed pregnancy, positive pregnancy test without a recorded miscarriage or abortion within the study period, birth, intra-uterine death or birth of a dead child after 20 weeks of gestation, breastfeeding or mastitis in combination with breastfeeding, or consultations regarding episiotomy). Women with charts indicating a pregnancy before January 1 2000, miscarriage or abortion before 20 weeks of gestation, recording of positive pregnancy test without the possibility of birth (e.g. because of restricted study time period or future pregnancy) and mastitis without mentioning of breastfeeding, were considered not to be pregnant.

All records identified as pregnancy were subsequently screened on history of PE. We considered a pregnancy as pre-eclamptic when a specified diagnosis of PE, or one of the more severe forms of PE, eclampsia or HELLP syndrome, were recorded in the EMD by the GP (or gynaecologist through correspondence with the GP). Eclampsia was defined as the occurrence of seizures in a pre-eclamptic patient. HELLP syndrome was defined to be a true case when this disorder was explicitly reported by the GP. Also women who in our opinion were strongly suspected to be PE cases according to the charts (e.g. by described combinations of hypertension and 'substantial' proteinuria) were selected. Of women who had more than one pregnancy during our follow-up observation period, we always used the information regarding the first pregnancy.

To compare cardiovascular risk factor management in the PE women with women whose pregnancy was uncomplicated, we randomly selected 150 women as control group.

### Collection of study population characteristics

Age of the women was determined at the time of delivery. Medical records were searched for signs of hypertension in a previous pregnancy, pre-existing vascular disease and chronic hypertension, because awareness of these conditions might influence cardiovascular risk management by the GP. Indications of hypertension already existing before pregnancy were the use of antihypertensive medication, multiple hypertensive blood pressure measurements or diagnosis recorded by the GP.

Follow-up time and the number and content of visits to the GP after pregnancy were determined, recording the frequency and timing of blood pressure, cholesterol and glucose measurements. Measurements obviously not related to the pregnancy or the pre-eclampsia, for example in case of fever, were excluded. Furthermore we screened on future (vascular) diagnoses by searching for specific ICPC codes (additional file 2) and vascular conditions or risk factors reported by the GP.

### Statistical analyses

Data are presented descriptively, providing number of women, mean values and standard deviations, or percentages. The differences between the PE cases and controls were investigated using *t*-test for continuous data, and χ^2^-test or Fisher exact test for categorical data as appropriate.

To verify the value of the ICPC coding for PE (W81, W81.1, W81.2, W81.3) we compared the PE codes with the information found in the records of all pregnant women. Subsequently, a 2 × 2 table was constructed and sensitivity, specificity, positive and negative predictive value were calculated. Data were analysed using SPSS for Windows (version 14.0, SPSS Inc., Chicago, Illinois, USA).

## Results

### ICPC and chart findings

Our search yielded 1196 women with 2232 pregnancies. Eighteen pregnancies (in eighteen women) were coded as having been complicated with a PE: W81 (n = 10), W81.1 (n = 1), W81.2 (n = 5) and W81.3 (n = 2). After examination of the charts, all patients were considered truly to have had pre-eclampsia. The review of the charts of the remaining 2214 pregnancies yielded another 17 patients diagnosed with PE, without a correct ICPC code. In total 35 patients experienced a PE (PE group), of which 2 developed eclampsia, and 5 developed the HELLP syndrome.

The ICPC coding for PE (combining W81, W81.1, W81.2, and W81.3) was calculated, showing a sensitivity of 51.4% and a specificity of 100.0%. The positive and negative predictive values were 100.0% and 99.2%, respectively.

### Patient characteristics

Characteristics of women with and without PE are given in Table 1.

**Table 1:** Characteristics of the pre-eclampsia group and controls

	PE group (n = 35)	Control group (n = 150)	*P*
Nullipara (%)	75.8	58.7	*0.07*^‡^
Mean age (y) ± SD	31.9 ± 3.6	32.4 ± 3.8	0.49^†^
Hypertension in previous pregnancy (%)	5.7	3.3	0.62^§^
Chronic hypertension (%)	14.3	4.0	0.02^‡^
Pre-existing vascular disease (%)	0.0	0.7	1.00^§^

NA, not applicable

^†^*t *test for independent samples

^‡ ^Chi-square test

^§^Fisher exact test

### Follow-up and risk factor management

Mean follow-up time was 2.9 years in the PE group and 2.5 years in the control group.

Blood pressure measurements were more often registered in women with a pregnancy complicated with PE than in women with an uncomplicated pregnancy (57.1% vs. 12.0% respectively, p < 0.001) (Table 2). The timing of the blood pressure measurements in the PE group varied. BP measurements were only found in the first 4 weeks postpartum in 35%, and up to 6 months in 25% of the women followed up.

**Table 2:** Risk factor management and cardiovascular diagnoses after pregnancy.

	PE Group (n = 35)	Control Group (n = 150)	*P*
Mean follow-up time (y) ± SD	2.9 (1.9)	2.5 (1.8)	0.29^†^
Blood pressure control	20 (57.1)	18 (12.0)	< 0.001^§^
Frequency			
*1 time*	*5 (25.0)*	*10 (55.6)*	
*2 times*	*3 (15.0)*	*4 (22.2)*	
≥ *3 times*	*13 (65.0)*	*4 (22.2)*	
Indication			
*PE/partus related*	*14 (70.0)*	*2 (11.8)*	
*Hypertension*	*6 (30.0)*	*5 (29.4)*	
*Complaints of vascular disease*	*0 (0.0)*	*5 (29.4)*	
*Screening/control*	*0 (0.0)*	*5 (29.4)*	
Timing			
*0-4 wks*	*7 (35.0)*	*3 (16.7)*	
*4-12 wks*	*4 (15.0)*	*0 (0.0)*	
*12-26 wks*	*5 (25.0)*	*0 (0.0)*	
*26-52 wks*	*0 (0.0)*	*0 (0.0)*	
≥ *52 wks*	*5 (25.0)*	*15 (83.3)*	
Glucose control	1 (3.0)	5 (3.3)	1.00^‡^
Cholesterol control	3 (9.1)	7 (4.7)	0.39^‡^
Cardiovascular diagnoses			
Hypertension*> 3 months postpartum	6/30**(20.0)	0 (0.0)	< 0.001^‡^
Hypercholesterolemia	0 (0.0)	1 (0.7)	1.00^‡^
Gestational hypertensive disorder	2 (6.1)	1 (0.7)	0.08^‡^
Cardio-/cerebrovascular disorder	1 (3.0)	3 (2.0)	1.00^‡^

Data reported as n (%) unless otherwise noted.

*≥1 measurement(s) of ≥140/90 mmHg

**Previously non-hypertensive women

^† ^Mann Whitney *U *test

^§ ^Chi-square test

^‡ ^Fisher exact test

The women in the PE group with an indication of chronic hypertension (n = 5) all had blood pressure measurements after delivery. In four of them these measurements were found only within three weeks, and within two, three and four months after delivery respectively.

In only 8 out of 30 women without signs of chronic hypertension the registration of blood pressure measurements continued after three months. Six of these women still had one or more hypertensive measurements after this period.

Of the 18 women who were coded by the GP as having had pre-eclampsia (W81, W81.1, W81.2, or W81.3), 61.1% had blood pressure measurements after delivery. In 54.5% of these women measurements took place within 3 months after delivery. For the women without the correct ICPC code, these numbers were 52.9% (Chi-square, p = 0.63) and 44.4% respectively (Fisher exact, p = 1.00).

In the PE group, the reason for the measurement recorded in the medical charts by the GP was in most cases because of the pre-eclampsia (70%), in the other women measurements were performed because of non-resolving hypertension (30%). In the control group various indications were recorded in the charts, including routine controls after birth, hypertension, complaints suspect for vascular disease such as chest pain, or screening for familial vascular diseases.

There were few glucose controls and cholesterol checks found in the medical records, and comparable between groups. Reasons for checking these risk factors recorded by the GP included screening for diabetes, familial high cholesterol or familiar vascular disease.

In the charts of 9 women of the PE group, one or more risk factors for vascular disease (e.g. obesity, diabetes, family history of cardiovascular disease and hypertension) were already present before the index pregnancy. After delivery, only two of these women were seen for these risk factors.

## Discussion

In this study we compared cardiovascular follow-up performed by GPs after pre-eclampsia and normotensive pregnancy. Fifteen out of the 35 women (42.9%) who experienced pre-eclampsia did not have blood pressure checks after pregnancy in their medical records. Glucose and cholesterol check ups were rare and similar among women with and without a PE.

### Strengths and limitations of the study

The strength of the current study lies in the fact that the data were obtained in a large community based cohort taken from primary practices, and in the suitability of the design to assess cardiovascular risk management after pre-eclampsia in routine and representative practice. However, the study had a small number of PE cases, which limits wide extrapolation of our findings due to the limited precision. Our study relied on the quality of the registration by the GPs. Although participating GPs were trained in working with electronic medical records, it might be possible that the GP did not report negative findings as often as positive ones, e.g. a normal blood pressure or negative family history, which would lead to a systematic underestimation of cardiovascular risk management by the GP. Some blood pressure check-ups might also not have been found in the medical records because they were measured by others then the GPs or by the patients themselves. In case of a pre-eclamptic pregnancy, women are normally invited for a blood pressure check-up six weeks after delivery. This check-up might be performed by the gynaecologist, the midwife or the GP. However, after this final control, women will not visit the gynaecologist nor midwife and will depend on their GP for blood pressure control.

In this study not all women with PE were coded as such by the GP. It could be hypothesized that GPs act differently with respect to follow-up when they explicitly code a pregnancy as pre-eclamptic. The number of women with blood pressure measurements after delivery and the timing of these measurements however, did not differ significantly between cases explicitly coded by the GP as PE, and cases without the correct ICPC code.

### Comparison with existing literature

Only a few studies, to our knowledge, have looked into maternity care after PE. In the study of Samwiil and co-workers [10], women with pregnancies complicated by pre-eclampsia were visited between six weeks and three months postpartum and were asked to recall if they had their blood pressure and urine tested at their six-week postnatal check. Of 257 audit participants across 21 maternity units in the United Kingdom, 91% recalled having had their blood pressure taken and 15% recalled having had their urine tested in general practices. In this study, 11% showed signs of unresolved pre-eclampsia. Macdonald and co-workers [11] evaluated the communication between maternity care providers and knowledge about the association between PE and future vascular disease via a survey. Only 54% of the participants were familiar with the long-term risks of hypertension after pre-eclampsia. Most care providers (83%) stated that they inform the patient's primary care physician about the hypertensive disorder in pregnancy, but few either informed the GP of the subsequent risk of hypertension (36%), or provided recommendations for follow-up (41%). Only 58% of the GPs reported that they are usually informed by the maternity care providers about their patients who developed a hypertensive disorder, and very few (12%) reported being informed regularly that such women are at risk for subsequently developing hypertension. The authors concluded that there are weaknesses in knowledge base and communication amongst maternity care providers that suggest that the identification and follow-up of women with hypertensive disorders of pregnancy is not occurring. Lack of knowledge could be a possible explanation for our finding that over 40% of the women who experienced pre-eclampsia never had a blood pressure check-up after pregnancy and that no more glucose and cholesterol check ups were performed compared with women who did not suffer from pre-eclampsia. It is possible that this was done by gynaecologists, however even if this is the case, it was not recorded in the patients medical charts. This might imply that GPs do not yet intensify their vascular risk policy towards women who experienced PE. Of note, in the Netherlands blood pressure measurements are not routinely taken when a woman visits her GP for other reasons.

### Validation ICPC coding

While ICPC codes are often used to estimate incidence or prevalence of certain health conditions or to select potential study objects, we are not aware of literature describing the validity of pre-eclampsia-related diagnoses collected in GPs registries. Our findings indicate that in the Utrecht Health Project ICPC codes for PE are correctly reported by the GP. Thus the use of ICPC codes can be recommended for etiologic studies into determinants of PE using a case control design. If the intention is to determine the incidence of PE, the sensitivity of ICPC codes is not accurate enough (missing over 50% of the cases).

There is no specific ICPC code for gestational hypertension only. Although the risk of future cardiovascular disease is lower for women who experienced gestational hypertension compared to women who experienced pre-eclampsia, the risk is still higher than for women who did not develop hypertension during pregnancy at all. Furthermore, the incidence of gestational hypertension is much higher compared to pre-eclampsia (about 10-15%). Probably, part of these women actually has chronic hypertension, mistaken for gestational hypertension due to the physiological fall in blood pressure that occurs early in pregnancy and the rise to or above the prepregnancy level in the second half of pregnancy. Because many young women have their first blood pressure measurements during pregnancy, ICPC coding for gestational hypertension might form a screening opportunity for chronic hypertension, which is under diagnosed in the Netherlands.

### Implications for future research or clinical practice

Our study showed a convincing number of patients in the PE group with probably ongoing or arising hypertensive disease after delivery. In at least six women hypertension existed longer then 3 months postpartum. In the other women follow-up did not last three months or blood pressure was not measured at all after pregnancy; the number of women with probably persistent (or chronic) hypertension is therefore likely to be higher.

Pregnancy may be considered as an important screening opportunity for cardiovascular and metabolic risk factors, identifying women who are at risk of cardiovascular disease later in life. Women might benefit from long term follow-up in terms of earlier diagnosis, intervention, counselling, life style changes and risk factor modification, with the ultimate aim to prevent future mortality and morbidity. Trial data on this issue is however lacking. Although it has been shown that women with a history of PE have an increased risk of not only chronic hypertension, but also of other cardiovascular conditions such as overweight and diabetes, the value of glucose and lipid measurement for identification of high risk groups is still unknown in this population. Furthermore, the 2007 update of the evidence-based guidelines for cardiovascular disease prevention in women by the American Heart Association states that future research should first evaluate the potential for medical contact during pregnancy to identify women at high risk. More research is needed on this aspect, and the necessity of development of screening and counselling guidelines deserves more attention and effort.

## Conclusion

In spite of the evidence of their increased risk of future cardiovascular disease, follow-up in women with a history of pre-eclampsia, is insufficient and undeveloped in primary care in the Netherlands, possibly due to a lack of knowledge and poor communication amongst obstetric and primary care providers.

## Competing interests

The authors declare that they have no competing interests.

## Authors' contributions

MEN reviewed the medical charts, performed the statistical analyses and drafted the manuscript. MT identified the ICPC codes, reviewed the medical charts and helped to draft the manuscript. AF, HB and DG advised on the interpretation of the data and revised the manuscript. MN is principal investigator of the Utrecht Health Study, under whose supervision data were collected, and he was involved in revising the manuscript. MB conceived of the study, participated in its design and helped to draft and write the manuscript. All authors critically revised the manuscript and approved the final report.

## Pre-publication history

The pre-publication history for this paper can be accessed here:

http://www.biomedcentral.com/1471-2296/10/77/prepub

## Supplementary Material

Additional file 1 **Appendix 1**. ICPC coding for conditions related to pregnancy Click here for file

Additional file 2 **Appendix 2**. ICPC codes used in additional search for vascular diagnoses and risk factors Click here for file
