# Supplementary material for: Cardiovascular risk factor assessment after pre-eclampsia in primary care
Source: BMC Fam Pract. 2009 Dec 8;10:77. doi: 10.1186/1471-2296-10-77 (PMC2796641; doi:10.1186/1471-2296-10-77)
Supplement: Additional file 1 — Appendix 1. ICPC coding for conditions related to pregnancy [file 1471-2296-10-77-S1.DOC]

**Appendix** 1. ICPC coding for conditions related to pregnancy

| W03 Antepartum bleeding  W05 Pregnancy vomiting/nausea  W17 Post-partum bleeding  W18 Post-partum symptom/complaint other  W19 Lactation symptom/complaint  W20 Breast symptom/complaint other  W27 Fear complications of pregnancy  W28 Limited function/disability related to pregnancy  W29 Other symptoms/complaints breast/pregnancy/puerperium  W70 Puerperal infection/sepsis  W71 Infection complicating pregnancy  W72 Malignant neoplasm related to pregnancy  W73 Benign/unspec. neoplasm/pregnancy  W75 Injury complicating pregnancy  W76 Congenital anomaly mother complicate pregnancy  W77 Non obstetric factors complicating pregnancy  W78 Pregnancy  W79 Unwanted pregnancy  W81 Toxaemia of pregnancy  W81.1 Pre-existent hypertension in pregnancy*  W81.2 Toxaemia/ pre-eclampsia*  W81.3 HELLP syndrome*  W84 Pregnancy high risk  W90 Uncomplicated labour/delivery livebirth  W91 Uncomplicated labour/delivery stillbirth  W92 Complicated labour/ delivery livebirth  W93 Complicated labour/delivery stillbirth  W94 Puerperal mastitis  W95 Breast disorder in pregnancy other  W96 Complications of puerperium other  W99 Disorder pregnancy/delivery other |
| --- |

* Subtitles are specific for The Netherlands (Dutch College of General Practitioners
