# Supplementary material for: Cardiovascular risk factor assessment after pre-eclampsia in primary care
Source: BMC Fam Pract. 2009 Dec 8;10:77. doi: 10.1186/1471-2296-10-77 (PMC2796641; doi:10.1186/1471-2296-10-77)
Supplement: Additional file 2 — Appendix 2. ICPC codes used in additional search for vascular diagnoses and risk factors [file 1471-2296-10-77-S2.DOC]

**Appendix 2.** ICPC codes used in additional search for vascular diagnoses and risk factors

| K74 Ischaemic heart disease w. angina  K75 Acute myocardial infarction  K76 Ischaemic heart disease w/o angina  K77 Heart failure  K85 Elevated blood pressure  K86 Hypertension uncomplicated  K87 Hypertension complicated  K89 Transient cerebral ischaemia  K90 Stroke/cerebrovascular accident  K91 Atherosclerosis  T82 Obesity  T83 Overweight |
| --- |
